# Relief After COVID-19 Vaccination: A Doubtful or Evident Outcome?

**DOI:** 10.3389/fmed.2021.800040

**Published:** 2022-01-10

**Authors:** Mohammed Khaled Al-Hanawi, Noor Alshareef, Rehab H. El-Sokkary

**Affiliations:** ^1^Faculty of Economics and Administration, Department of Health Services and Hospital Administration, King Abdulaziz University, Jeddah, Saudi Arabia; ^2^Health Economics Research Group, King Abdulaziz University, Jeddah, Saudi Arabia; ^3^Faculty of Medicine, Medical Microbiology and Immunology Department, Zagazig University, Zagazig, Egypt

**Keywords:** vaccine-related anxiety, trust, immunization program, COVID-19, relief, Saudi Arabia

## Abstract

**Background:** Since development of the first COVID-19 vaccine, the landscape of public confidence in these vaccines is uncertain. Building confidence is crucial for better preparedness of future pandemics. Following the mandatory COVID-19 vaccination policy in the country, the aim of this study was to examine whether the Saudi public feels relieved post-vaccination and to identify the factors predicting such relief.

**Methods:** An online cross-sectional survey was conducted in July 2021 among COVID-19 vaccine recipients in Saudi Arabia. A multivariable logistic regression analysis was employed to examine and identify the variables associated with feeling relieved post-vaccination.

**Results:** Most of the respondents (66%) stated feeling more relieved post-vaccination. Male gender [adjusted odds ratio (AOR): 1.380; 95% confidence interval (CI): 0.981–1.943], being a student (AOR: 3.902; 95% CI: 1.674–9.096), and received two doses of the vaccine (AOR: 2.278; 95% CI: 1.630–3.182) were associated with feeling more relieved after getting vaccinated. Respondents who were anxious about the vaccine before receiving it (AOR: 0.220; 95% CI: 0.160–0.302), and experienced a severe reaction after vaccination (AOR: 0.288; 95% CI: 0.165–0.504) had lower odds of feeling relieved post-vaccination. Respondents who relied on social media as the main source of vaccine-related information and those having no information about the vaccine were also less likely to feel relieved post-vaccination.

**Conclusions:** Individuals' attitudes toward COVID-19 vaccines may not necessarily alter post-vaccination. Although mandatory vaccination policies can significantly contribute to achieving herd immunity, public confidence toward vaccines might be eroded, which could in turn impose significant challenges in future pandemics efforts.

## Introduction

It is well-recognized that mass immunization efforts represent a critical step toward combating the COVID-19 pandemic (1). The development of COVID-19 vaccines has shown the world how substantial funding, research collaboration, and diligence can stimulate innovation to address public needs at a global level and in a short period of time (2). By early March 2021, stringent regulatory agencies worldwide started to authorize several highly effective vaccines. Nevertheless, the challenge of a vaccine-based solution to the COVID-19 pandemic does not end with the development of an effective and safe vaccine, as achieving herd immunity will require the vaccination of a significant proportion of the population (1). Despite the devastating impact COVID-19 has had on people's health and well-being, many remain unwilling to vaccinate against the virus. The wide spread of misinformation, coupled with mistrust in pharmaceutical companies (3–5) and concerns surrounding the safety and efficiency of vaccines (6–8) have been shown to undermine vaccine acceptance (2). Thus, enhancing public trust in COVID-19 vaccines and vaccination in general is as critical as developing effective vaccines (2).

The success of vaccination campaigns is dependent on peoples' trust in the effectiveness and safety of the vaccines, the competence and reliability of the delivering institutions, and the principles and processes underlying government decisions and actions (2). In December 2020, the World Health Organization (WHO) released a safety surveillance manual that provided guidance on many aspects of vaccine global efforts, highlighting the importance of adequate advance planning of immunization campaigns and how to support vaccine safety communication during the COVID-19 pandemic (9). Anxiety-related events that occur soon after COVID-19 vaccination may cause concern among other vaccine recipients and staff members, particularly in a mass vaccination scenario (10). When adverse events were reported related to the 2009 H1N1 influenza pandemic vaccine, some countries did not provide timely information about the association of the events with the vaccine, causing lack of confidence in the vaccine that subsequently influenced vaccine uptake and communication (11, 12). Thus, sustaining trust during a pandemic is critical for health authorities, both if repeated vaccinations are necessary and in preparation for future health emergencies (13).

Willingness to become vaccinated changes over time, and those hesitating are likely to be taking their cues from others (10). For example, a study conducted in the USA showed that 69% of individuals who had not been vaccinated but lived in a household with someone who had already been vaccinated were willing to vaccinate against COVID-19 as soon as possible; this was also true among those who were not vaccinated but had a close friend or family member outside their households who already received a vaccine. By contrast, only one third of individuals who had a causal connection or no connection to a vaccinated person were willing to receive the vaccine as soon as possible (14). It is suggested that as more individuals are vaccinated, more will be willing to accept vaccination (2). This may reflect that being a recipient of the vaccine gradually becomes the norm and is recognized as the way out of confinement and restriction. Moreover, conformism or “herd behavior” might have a positive influence on the diffusion of vaccination even if population acceptance of the vaccine is modest, which can be attributed to the fact that individuals who were initially wiling to vaccinate may send a positive signal about their beliefs of the safety and efficacy of the vaccine (10).

A survey assessing the acceptability of COVID-19 vaccines among the Saudi population prior to arrival of the vaccine to the country showed that only 48% of respondents were willing to be vaccinated (15). Another public survey conducted after the rollout of national vaccination program in the country showed an acceptance rate of 60% (16). As the time of this study, report shows that around 70% of the population received the COVID-19 vaccine in Saudi Arabia (17). Following mandatory vaccination policies in the country and elsewhere, this study investigated how people feel after receiving the vaccine. Given that individuals' experience with COVID-19 will shape their confidence in other vaccines, and to help promote confidence at this time, it is critical to understand if people feel relieved after receiving the COVID-19 vaccine. Therefore, the objective of this study was to examine whether vaccinations made the general Saudi public feel more relieved and to identify the factors predicting such relief. This can shed light on future efforts needed when novel vaccines are introduced in response to pandemics.

## Materials and Methods

### Study Design and Sample

This study used an online cross-sectional survey conducted between 13 and 20 July 2021 among adults aged 18 years or older, who were vaccinated against COVID-19 (one or two doses) and living in the Kingdom of Saudi Arabia (KSA). Participants were asked to participate in a self-administered online questionnaire using SurveyMonkey Inc. (San Mateo, CA, USA). Invitations to participate in the study were distributed to respondents via social media platforms, including Twitter (San Francisco, CA, USA) and WhatsApp Inc. (Mountain View, CA, USA). A simplified snowball sampling technique was used to recruit participants. The online approach was being used to avoid any physical contact. Moreover, an online approach of data collection via social media platforms has been used in several studies during the COVID-19 pandemic, and can be considered, to a great extent, as a credible approach (6, 18, 19).

We aimed to maximize reach and gather data from as many respondents as possible. According to the latest KSA census, Saudi Arabia has a population of 35,013,414 (20). Using a sample size calculator (21), the representative target sample size was calculated to be 1037 participants, using a margin of error of ±4%, confidence level of 99%, 50% response distribution, and 35,013,414 people.

Online informed consent was obtained from all participants before proceeding with the questionnaire. The respondents were clearly informed about the aim and objectives of the study, their voluntary participation and their right to withdraw from the study at any time, and that all information would be anonymous and confidential. A total of 1,094 participants completed the questionnaire across the 13 regions in the KSA. After excluding responses from participants living outside the KSA and those with missing data on variables of interest, the final sample for analysis comprised responses from 1,058 participants.

The self-reported questionnaire was designed and developed based on similar studies and frameworks to assess potential post-vaccination side effects and stress-related information following immunization (22–25). The questionnaire was validated by an expert panel who carefully reviewed the items of the questionnaire and provided feedback and suggestions. The questionnaire was then updated based on their feedback and suggestions. The reliability of the questionnaire was also evaluated using the Cronbach alpha test. Cronbach's alpha coefficient was 0.75, indicating good internal reliability (26).

The questionnaire consisted of four main sections. The first section gathered information on the respondents' sociodemographic characteristics. The second section gathered information on health behaviors, type of vaccination received, and knowledge and sources of information about COVID-19 vaccines. The third section gathered information related to perceptions and beliefs following vaccination. The final section collected information on the reaction after vaccination. The questionnaire was originally written in English and then translated into Arabic. The survey was administered in Arabic.

### Measures

#### Outcome Measure

The main outcome variable for this study was relief after receiving the COVID-19 vaccine. To measure relief, participants were asked if they felt relieved after vaccination. The responses to this question were “yes” or “no.” This outcome variable was coded as a binary variable, with 0 for those stating not feeling relieved after taking the vaccine and 1 for those stating feeling relieved following vaccination.

#### Explanatory Variables

Some explanatory variables were collected and assessed to place the results in context. Respondents were asked about their sociodemographic characteristics, including age, gender, marital status, educational level, and employment status. The age variable was divided into five categories: 18–29 (reference category), 30–39, 40–49, 50–59, and ≥60 years. Gender was coded as a binary variable, with 1 for male and 0 for female. Marital status was also captured as a binary variable, with 1 for married and 0 for unmarried (including single, widowed, and divorced). Educational level was divided into three categories: high school or below (reference category), university degree, and postgraduate degree. Employment status was categorized into four groups: government employee (reference group), private sector employee, students, unemployed, and retired.

Respondents were also asked whether they suffer from chronic illness, were smokers, had been infected with COVID-19 before vaccination, were concerned about the COVID-19 vaccine before taking it, their main source of information about COVID-19 vaccines, the type of vaccine received, number of doses received so far, had a COVID-19 breakthrough infection, and if they had any side effects after getting the vaccine.

### Statistical Analyses

Bivariate and multivariable regression analyses were the major analytical tools employed in this study. Bivariate analysis of categorical variables was performed using chi-squared tests to determine the associations between our dependent variable of interest (feeling more relieved after vaccination) and independent variables. A multivariable logistic regression analysis was employed to examine and identify the variables associated with feeling relieved post- vaccination, with the adjusted odds ratio (AOR) and 95% confidence interval (CI) calculated. All analyses were conducted using STATA 16.1 software (StataCorp LP, Texas, USA).

### Ethical Considerations

All procedures performed in this study involving human participants complied with the institutional and/or national research committee ethical standards and the 1964 Helsinki Declaration and subsequent amendments or equivalent ethical standards. This research has been reviewed and given a favorable opinion by King Abdulaziz University. The study was designed and conducted according to the ethical principles established by King Abdulaziz University. Therefore, ethical approval was obtained from the Biomedical Ethics Research Committee, Faculty of Medicine, King Abdulaziz University (Ref-380-21).

## Results

Table 1 presents the characteristics of participants and the potential factors influencing the feeling of relieved after COVID-19 vaccination. Among the 1,058 respondents, 703 (66.45%) felt relieved after receiving the vaccine. Most of the respondents were aged below the age of 49 years, married, and had higher education. Approximately half of the respondents were female and employed in the government sector.

**Table 1:** Frequency distribution and chi-square analysis of post-COVID-19 vaccination relief (*N* = 1,058).

**Variable**	**Not feeling relieved after vaccination** ***n*** **=** **355**		**Feeling relieved after vaccination** ***n*** **=** **703**		**Total (%)**		***P*-value**
	***n***	**%**	***n***	**%**	***n***	**%**	
**Age (years)**							
18–29	54	15.21	89	12.66	143	13.52	0.003^***^
30–39	135	38.03	228	32.43	363	34.31	
40–49	103	29.01	191	27.17	294	27.79	
50–59	44	12.39	110	15.65	154	14.56	
≥60	19	5.35	85	12.09	104	9.83	
**Gender**							
Female	223	66.82	317	45.09	540	51.04	<0.001^***^
Male	132	37.18	386	54.91	518	48.96	
**Marital status**							
Unmarried	91	25.63	171	24.32	262	24.76	
Married	264	74.37	532	75.68	796	75.24	0.641
**Educational level**							
High school or below	61	17.18	168	23.90	229	21.64	0.015^**^
University degree	173	48.73	287	40.83	460	43.48	
Postgraduate degree	121	34.08	248	35.28	369	34.88	
**Employment status**							
Government employee	181	50.99	360	51.21	541	51.13	<0.001^***^
Private sector employee	65	18.31	96	13.66	161	15.22	
Students	15	4.23	55	7.82	70	6.62	
Unemployed	19	5.35	91	12.94	110	10.40	
Retired	75	21.13	101	14.37	176	16.64	
**Suffer from** **chronic illness**							
No	243	68.45	462	65.72	705	66.64	0.373
Yes	112	31.55	241	34.28	353	33.36	
**Smoker** **(cigarettes or shisha)**							
No	288	81.13	510	72.55	798	75.43	0.002^***^
Yes	67	18.87	193	27.45	260	24.57	
**Infected with COVID-19** **before vaccination**							
No	301	84.79	605	86.06	906	85.63	0.578
Yes	54	15.21	98	13.94	152	14.37	
**Feeling anxious about the COVID-19** **vaccine before receiving it**							
No	78	21.97	425	60.46	503	47.54	<0.001^***^
Yes	277	78.03	278	39.54	555	52.46	
**Main source of information** **about COVID-19 vaccines**							
Official media/scientific or medical websites	243	68.45	556	79.09	799	75.52	<0.001^***^
Social media platforms	60	16.9	73	10.38	133	12.57	
Friends and relatives	21	5.92	45	6.40	66	6.24	
Have no information	31	8.73	29	4.13	60	5.67	
**Number of doses** **received so far**							
One	234	65.92	304	43.24	538	50.85	<0.001^***^
Two	121	34.08	399	56.76	520	49.15	
**Had a COVID-19** **breakthrough infection**							
No	324	91.27	653	92.89	977	92.34	0.349
Yes	31	8.73	50	7.11	81	7.66	
**Had reaction** **to the vaccine**							
No side effects at all	69	19.44	189	26.88	258	24.39	<0.001^***^
Mild side effects	138	38.87	314	44.67	452	42.72	
Moderate side effects	92	25.92	153	21.76	245	23.16	
Severe side effects	56	15.77	47	6.69	103	9.74	

*** *P < 0.01,*

** *P < 0.05*.

One-third of the respondents were suffering from a chronic illness and about one-quarter were smokers. More than half of the participants indicated being anxious about the vaccine before receiving it. Approximately three-quarters of the participants indicated that the official media and scientific or medical websites were their main sources of information about COVID-19 vaccines. About half of the respondents had received two doses of the vaccine, while only 7% stated that they became infected with COVID-19 after vaccination (i.e., “breakthrough” infection). Approximately 10% of the respondents indicated that they had severe side effects after receiving the vaccine. As shown in Table 1, age, gender, information source about the vaccine, number of doses received, and having side effects after receiving the vaccine were significantly associated with feeling more relieved post-vaccination at the 1% level.

Table 2 presents the results of logistic regression estimates of factors associated with feeling relieved after COVID-19 vaccination. With increasing age, the participants felt more relieved. Comparing to females, male respondents felt more relieved after vaccination (AOR: 1.380; 95% CI: 0.981–1.943). Although there were no significant associations with most of the employment status categories, being a student was associated with feeling relieved after vaccination (AOR: 3.902; 95% CI: 1.674–9.096). Compared to those who were not initially anxious about the vaccine before vaccination, participants who reported being anxious were still not feeling relieved post vaccination (AOR: 0.220; 95% CI: 0.160–0.302).

**Table 2:** Logistic regression estimates of factors associated with feeling relieved after COVID-19 vaccination (*N* = 1,058).

**Variable**	**AOR**	**95% CI**	***p*-value**
**Age (years)**			
18–29 (ref)			
30–39	1.668	0.914–3.043	0.096^*^
40–49	1.712	0.912–3.213	0.094^*^
50–59	2.062	0.998–4.260	0.051^*^
≥60	2.007	0.800–5.033	0.138
**Gender**			
Female (ref)			
Male	1.380	0.981–1.943	0.064^*^
**Marital status**			
Unmarried (ref)			
Married	0.905	0.608–1.349	0.625
**Educational level**			
High school or below (ref)			
University degree	0.667	0.443–1.003	0.051^*^
Postgraduate degree	0.712	0.452–1.121	0.142
**Employment status**			
Government employee (ref)			
Private sector employee	0.734	0.473–1.141	0.170
Students	3.902	1.674–9.096	0.002^***^
Unemployed	1.124	0.533–2.373	0.758
Retired	1.240	0.781–1.968	0.361
**Suffer from** **chronic illness**			
No (ref)			
Yes	1.070	0.760–1.506	0.700
**Smoker** **(cigarettes or shisha)**			
No (ref)			
Yes	1.284	0.882–1.870	0.193
**Infected with COVID-19** **before vaccination**			
No (ref)			
Yes	1.089	0.708–1.675	0.698
**Feeling anxious about the** **COVID-19 vaccine before receiving it**			
No (ref)			
Yes	0.220	0.160–0.302	0.000^***^
**Main source of information** **about COVID-19 vaccines**			
Official media/scientific or medical websites (ref)			
Social media platforms	0.569	0.371–0.875	0.010^**^
Friends and relatives	0.839	0.456–1.544	0.573
Have no information	0.408	0.221–0.752	0.004^***^
**Number of doses** **received so far**			
One (ref)			
Two	2.278	1.630–3.182	0.000^***^
**Had a COVID-19** **breakthrough infection**			
No (ref)			
Yes	0.977	0.565–1.689	0.934
**Had reaction to the vaccine**			
No side effects at all (ref)			
Mild side effects	0.848	0.572–1.257	0.411
Moderate side effects	0.595	0.382–0.926	0.021^**^
Severe side effects	0.288	0.165–0.504	0.000^***^

*** *P < 0.01*.

** *P < 0.05*.

* *P < 0.1*.

Another interesting result was that respondents who relied on social media as the main source of information about the vaccine and those having no information about the vaccine had lower odds of feeling more relieved after vaccination compared with those who relied on official sources and scientific or medical websites. Respondents who received two doses of the vaccine were two times more likely to feel relieved after getting the vaccine compared with those who had received only one dose (AOR: 2.278; 95% CI: 1.630–3.182). Lastly, those who experienced severe side effects after vaccination had lower odds of feeling more relieved after vaccination compared with those who did not have any side effects at all (AOR: 0.288; 95% CI: 0.165–0.504).

## Discussion

For COVID-19 vaccine efforts to be successful, the safety and efficacy of the vaccine as well as its wide acceptance should be ensured. To increase public resilience to any myths and rumors surrounding the vaccine and to strengthen further support for vaccination programs, it is important to understand whether or not people feel relieved after receiving the COVID-19 vaccine. Thereby, the aim of this study was to examine post-vaccination relief among the general Saudi population and the contributing factors. It is important to identify factors of relief after being vaccinated to help guide recommendations for encouraging vaccination and preventing the further spread of COVID-19. We found that males, older participants, and those who completed the two-dose vaccination series felt relieved after COVID-19 vaccination. Participants relying on social media as the main source of information about the COVID-19 vaccine and those experiencing serious adverse side effects were less likely to feel relieved post-vaccination.

Studies assessing vaccination acceptance reported that older adults are more likely to accept the COVID-19 vaccine (27). It is thus not surprising that our results showed that as the age of participants increased, the feeling of relief after receiving the vaccine became more common. This study also showed gender differences in feeling relieved about the vaccine post-vaccination, with males being more likely to be relieved post-vaccination than females. This finding could be due to the fact that males are more likely to be infected with COVID-19 (28) and are less likely to engage in preventive behavior than females (29, 30).

Inconsistent with previous studies, the results showed that the participants' anxiety about the vaccine was not eased even after being vaccinated (25). Although clinical trials support the safety and efficacy of the Oxford-AstraZeneca and Pfizer vaccines, both of which were commonly used in the KSA during the study period (31, 32), reported cases of thrombosis and thrombocytopenia syndrome that occurred following immunization with the Oxford-AstraZeneca vaccine (33, 34) could have provoked anxiety and eroded public confidence. Post-vaccination side effects might have also further contributed to feelings of anxiety post-vaccination. Thereby, informing the public on what to expect after vaccination can help in dispelling myths and lowering anxiety about the vaccine (35). Two-way communication that allows and encourages the public to ask questions could ensure that actual rather than assumed questions are adequately addressed (36).

Additionally, in countries such as the KSA where vaccination is mandatory, feelings of anxiety might not necessarily be eased post-vaccination. One plausible reason is that enforcement undermines faith in the government while also signaling that the government does not trust the individual to uphold the social norm of protecting others (37). As a result, the distrust expressed by law enforcement implies low expectations of peoples' behavior. From the perspective of citizens, this might lead to a mutually distrustful relationship (38, 39) that promotes vaccination reluctance. Furthermore, enforcement may diminish the beneficial conformism-based impacts of others who have been vaccinated. This is because individuals who initially agree to get vaccinated may convey a favorable signal about their desire to collaborate as well as their trust in the vaccine's safety and effectiveness. Conversely, compliance with mandated vaccination conveys a considerably weaker signal. However, if vaccination desire is inadequate to generate cumulative gains in compliance, enforcement will be necessary (10).

Unsurprisingly, the results also showed that people who sourced their COVID-19 vaccine information from social media were less likely to feel relieved post-vaccination. Uncertainty and doubts fueled by rumors and myths surrounding vaccines link to infertility, harm, and altering the DNA can build on feelings of discomfort and anxiety post-vaccination (40).

The main limitations of this study include the use of an online survey that might have impacted the study's generalizability, the cross-sectional design, and lack of data on non-respondents. The results are only applicable to this context and may differ among other populations due to different vaccine policies, laws, and regulations. The social distancing regulations favor the use of online platforms in data collection, however using snowball sampling technique via social media platforms would have affected the representativeness of the sample. Moreover, this study does not imply causality, given that no causal identification methods were employed for analysis. A further limitation is that we have not examined the source of feeling “unrelieved,” nor have we studied the psychological components which might have impacted these feelings.

## Conclusion

Although safe and effective COVID-19 vaccines are now being administered across the KSA along with announcements that vaccines are 95% effective, doubts and concerns surrounding vaccination remain a challenge. Knowledge about people's experience post-vaccination in the real world remains scarce. Our study disclosed that most of the Saudi population (around 66%) feel relieved after receiving the mandatory COVID-19 vaccine. We found that female gender, relying on social media for information concerning COVID-19 vaccines, and feeling anxious about the vaccine prior to vaccination were associated with a lack of relief post-vaccination. Individuals' attitudes toward COVID-19 vaccines may not necessarily alter even post-vaccination. Although mandatory vaccination policies can significantly contribute to achieving herd immunity, public confidence toward vaccines might be eroded and might in turn impose significant challenges in future pandemic efforts.

## Data Availability Statement

The datasets generated and/or analyzed during the current study are not publicly available due to privacy and confidentiality agreements as well as other restrictions but are available from the corresponding author on reasonable request.

## Ethics Statement

All procedures performed in this study involving human participants complied with the institutional and/or national research committee ethical standards and the 1964 Helsinki Declaration and subsequent amendments or equivalent ethical standards. This research has been reviewed and given a favorable opinion by King Abdulaziz University. The study was designed and conducted according to the ethical principles established by King Abdulaziz University. Therefore, ethical approval was obtained from the Biomedical Ethics Research Committee, Faculty of Medicine, King Abdulaziz University (Ref-380-21). The participants provided their online informed consent to participate in this study.

## Author Contributions

MA-H: conceptualization, data curation, formal analysis, methodology, project administration, software, supervision, and funding acquisition. MA-H, NA, and RE-S: validation, writing—original draft preparation, writing—review, and editing. All authors have read and agreed to the published version of the manuscript.

## Funding

This research was funded by the Institutional Fund Projects under grant number IFPRC-207-121-2020. The funders had no role in the study design, data collection and analysis, decision to publish, or preparation of the manuscript.

## Conflict of Interest

The authors declare that the research was conducted in the absence of any commercial or financial relationships that could be construed as a potential conflict of interest.

## Publisher's Note

All claims expressed in this article are solely those of the authors and do not necessarily represent those of their affiliated organizations, or those of the publisher, the editors and the reviewers. Any product that may be evaluated in this article, or claim that may be made by its manufacturer, is not guaranteed or endorsed by the publisher.
